# The Effects of 16 Weeks of Exercise Training on Neutrophil Functions in Breast Cancer Survivors

**DOI:** 10.3389/fimmu.2021.733101

**Published:** 2021-10-27

**Authors:** David B. Bartlett, Erik D. Hanson, Jordan T. Lee, Chad W. Wagoner, Elizabeth P. Harrell, Stephanie A. Sullivan, Lauren C. Bates, Mohamdod S. Alzer, Dean J. Amatuli, Allison M. Deal, Brian C. Jensen, Grace MacDonald, Michael A. Deal, Hyman B. Muss, Kirsten A. Nyrop, Claudio L. Battaglini

**Affiliations:** ^1^ Division of Medical Oncology, Duke Cancer Institute, Duke University, Durham, NC, United States; ^2^ Duke Molecular Physiology Institute, Duke University, Durham, NC, United States; ^3^ Department of Nutritional Sciences, Faculty of Health and Medical Sciences, University of Surrey, Guildford, United Kingdom; ^4^ Department of Exercise and Sport Science, University of North Carolina at Chapel Hill, Chapel Hill, NC, United States; ^5^ Human Movement Science Curriculum, University of North Carolina at Chapel Hill, Chapel Hill, NC, United States; ^6^ Lineberger Comprehensive Cancer Center, University of North Carolina at Chapel Hill, Chapel Hill, NC, United States; ^7^ Division of Cardiology, University of North Carolina at Chapel Hill, Chapel Hill, NC, United States; ^8^ Department of Medicine, University of North Carolina, Chapel Hill, NC, United States

**Keywords:** breast cancer survivors, innate immune function, exercise training, neutrophils, acute exercise

## Abstract

Following therapy, breast cancer survivors (BCS) have an increased risk of infections because of age and cancer dysregulation of inflammation and neutrophil functions. Neutrophil functions may be improved by exercise training, although limited data exist on exercise and neutrophil functions in BCS.Sixteen BCS [mean age: 56 (SD 11) years old] completed 16 weeks of community-based exercise training and a 45-minute acute bout of cycling before (Base) and after (Final) the exercise training program. Exercise training consisted of 3 x 40 – 60 minute mixed mode aerobic exercises, comprising 10 – 30 minutes aerobic and 30 minutes resistance training. At Base and Final, we took BCS blood samples before (PRE), immediately after (POST), and 1 hour after (1Hr) acute exercise to determine neutrophil counts, phenotype, bacterial killing, IL-6, and IL-8 levels. Eleven healthy, age- and physical activity levels-matched women (Control) completed the acute bout of exercise once as a healthy response reference. Resting Responses. BCS and Controls had similar Base PRE absolute neutrophil counts [mean (SD): 3.3 (1.9) v 3.1 (1.2) x 10^9^/L, p=0.801], but BCS had lower bacterial phagocytosis [3991 (1233) v 4881 (417) MFI, p=0.035] and higher oxidative killing [6254 (1434) v 4709 (1220) MFI, p=0.005], lower CD16 [4159 (1785) v 7018 (1240) MFI, p<0.001], lower CXCR2 [4878 (1796) v 6330 (1299) MFI, p=0.032] and higher TLR2 [98 (32) v 72 (17) MFI, p=0.022] expression, while IL-6 [7.4 (5.4) v 4.0 (2.7) pg/mL, p=0.079] levels were marginally higher and IL-8 [6.0 (4.7) v 7.9 (5.0) pg/mL, p=0.316] levels similar. After 16 weeks of training, compared to Controls, BCS Final PRE phagocytosis [4510 (738) v 4881 (417) MFI, p=0.146] and TLR2 expression [114 (92) v 72 (17) MFI, p=0.148] were no longer different. Acute Exercise Responses. As compared to Controls, at Base, BCS phagocytic Pre-Post response was lower [mean difference, % (SD): 12% (26%), p=0.042], CD16 Pre-Post response was lower [12% (21%), p=0.016] while CD16 Pre-1Hr response was higher [13% (25%), p=0.022], TLR2 Pre-Post response was higher [15% (4%) p=0.002], while IL-8 Pre-Post response was higher [99% (48%), p=0.049]. As compared to Controls, following 16 weeks of training BCS phagocytic Pre-Post response [5% (5%), p=0.418], CD16 Pre-1Hr response [7% (7%), p=0.294], TLR2 Pre-Post response [6% (4%), p=0.092], and IL-8 Pre-Post response [1% (9%), p=0.087] were no longer different. Following cancer therapy, BCS may have impaired neutrophil functions in response to an acute bout of exercise that are partially restored by 16 weeks of exercise training. The improved phagocytosis of bacteria in BCS may represent an exercise-induced intrinsic improvement in neutrophil functions consistent with a reduced risk of infectious disease.

**Clinical Trial Registration:**

ClinicalTrials.gov, identifier NCT03760536.

## 1 Introduction

Recent advances in breast cancer detection and treatment have increased 5-year survival rates to approximately 90% (1). However, following the completion of therapy, breast cancer survivors (BCS) suffer prolonged immune suppression, increasing the risk of recurrence, metastasis, and microbial infections (2, 3). Neutrophils are the first immune cell to respond against microorganisms and are functionally suppressed during and after chemotherapy and surgery (4, 5). Neutrophil functional responses are stimulated by inflammatory cytokines, including IL-6 and IL-8 (6). However, chronic exposure to inflammation, as seen in breast cancer, can generate neutrophils with suppressed microbial functions (7). Therefore, interventions that improve BCS neutrophil functions may reduce the risk of infections following the completion of therapy.

In the general population, exercise training and increasing physical activity are associated with improved immune functions, including neutrophils (8, 9). Although there is a growing body of evidence for exercise-mediated immune improvements in other cancers (10), in BCS, immune function changes following exercise training are less clear (10–12). Although NK-cell cytotoxicity and lymphocyte proliferation increased following 4 and 6 months of exercise training, respectively (13, 14), other studies show no effects of exercise training (15). The only BCS exercise study assessing neutrophils we are aware of observed no impact on mitogen-stimulated neutrophil oxidative burst or granularity (13). However, our recent data in non-cancer adults suggests that with increased physical activity and exercise training, neutrophil bactericidal functions are improved (16–19). Neutrophil bactericidal assays represent a physiologically relevant stressor to assess neutrophils’ intrinsic physiologic reserve (20). However, the neutrophil functional reserve is most important upon initiation of a pathogenic and inflammatory challenge. Therefore, to assess functional reserve capacity better, a physiological stressor that mimics a pathogenic challenge is required. Although the pathophysiology is considerably different (i.e. no fever from exercise), there are similarities between a pathogenic challenge and an acute bout of exercise. Specifically, both result in a transient increase in white blood cells, including neutrophils, and both have a transient increase in IL-6 and IL-8, that all return to normal upon resolution of infection or completion of exercise. The latter can be considered a safe and sterile experimental stressor that characterizes immunologic reserve (8, 21).

The acute exercise immune response in healthy adults is well characterized for major leukocyte subsets (8, 22), and there are a growing number of acute exercise studies in BCS (23, 24). For the first time that we are aware of, we have recently described in BCS the acute exercise-mediated monocyte (25) and mucosal-associated invariant T-cell (MAIT) (26) responses before and after 16 weeks of exercise training. Our results suggest that the acute response was beneficially modulated following 16 weeks of exercise training. As such, understanding the BCS ‘acute stress’ immune response concerning bacterial infections, and how exercise training modulates responses will provide needed insight into exercise prescription for BCS to improve their immunological responses against commonly encountered pathogenic stressors.

Therefore, this study aimed to determine whether the neutrophil and inflammatory responses to an acute physiological stressor (i.e., a single bout of exercise) differed between BCS and healthy women, and if 16 weeks of exercise training altered the acute neutrophil response of BCS. Specifically, we assessed whether neutrophil phagocytosis and oxidative killing of bacteria, and cell surface receptor expression of neutrophil functional markers (i.e., CD16, CXCR2, TLR2, and TLR4), and circulating IL-6 and IL-8 were altered.

## 2 Methods

The study design and methods have been described in detail previously and consisted of mostly the same women in our previous studies (16, 18, 19, 25, 26). Briefly, all participants were physically inactive and a sub-set from a more extensive investigation (NCT03760536). Sixteen BCS (previously diagnosed with stage I-III breast cancer) and eleven healthy women (Control) matched for age, and physical activity levels completed the study. Participants were inactive, defined as exercising <30 minutes at a moderate intensity twice per week that failed to meet guidelines established by the American College of Sports Medicine. As the neutrophil component was a secondary objective and relied on additional support, all samples were acquired depending on availability of the neutrophil team. For this sub-study all women completed the pre-planned neutrophil physical assessments. Participants provided written informed consent, and the study was approved by the Protocol Review Committee and the Institutional Review Board at the University of North Carolina at Chapel Hill which conforms to the Declaration of Helsinki.

### 2.1 Study Design

Briefly, BCS and Controls completed baseline (Base) testing before BCS enrolled in a 16 week community-based aerobic and resistance training program that consisted of three sessions/week for ~1 hour per session. After training, follow-up testing (Final) for BCS was the same as Base. Testing visits included a preliminary assessment and familiarization, maximal cardiopulmonary exercise testing and body composition, and an acute bout of exercise at 60% of peak power with blood taken before exercise (Pre), immediately following completion of exercise (Post) and 1 hour after exercise completion (1Hr Post) (25, 26). **Table 1** shows the BCS exercise training prescription and their adherence and compliance to the program with this sub-study utilizing additional participants from our previous publications.

**Table 1:** Exercise Prescription and actual exercise completion by BCS.

	Prescribed			
Total Sessions/week (N)	3			
Time per Session (min)	40 - 60			
Total Sessions (N)	48			
Exposure (mins/week)	154 - 180			
	**Aerobic**		**Resistance**	
	**Intensity**	Duration (mins/session)	**Intensity**	Duration (mins/session)
	Week 1 – 2 RPE: 8-11; Low Intensity	10-15	Week 1 – 2 RPE: 7-13; Low – Mod Intensity 1 set/15 reps	30
	Week 3 – 7 RPE: 8-11; Low Intensity	10 – 30	Week 3 – 5 RPE: 7-13; Low – Mod Intensity 2 sets/10-30 reps	30
	Week 8 – 16 RPE 12 – 14; Mod Intensity	30	Week 6 – 16 RPE: 14-15; High Intensity 2 sets/10 reps	30
**Actual Completion**				
Adherence Number of days attended Percentage of prescribed days attended	35.6 (10.6) 74.2% (22.0%)			
	**Aerobic**	**Resistance**		
Compliance for Prescribed Workload Number of sessions completed Percentage of prescribed sessions	24.3 (11.2) 50.7% (23.4%)	12.9 (5.5) 26.8% (11.5%)		

RPE, ratings of perceived exertion; Mod: Moderate, Data are mean (SD).

### 2.2 Testing Visits

#### 2.2.1 Visit 1: Preliminary Assessments and Familiarization

During Visit 1, participants completed a medical history and a familiarization of the exercise test. The medical screening included a resting 12-lead electrocardiogram that was reviewed by the study cardiologist for clearance to partake in the exercise program. Participants then performed a familiarization session on an electronically braked cycle ergometer that included being fitted for the ergometer (Lode electronically braked cycle ergometer (Lode, Groningen, The Netherlands) and the mask (Hans Rudolph, Shawnee, KS). Participants wore a heart rate monitor (Polar Electro Inc., Lake Success, NY) chest strap, and heart rate (HR) was recorded continuously during each exercise visit. After resting quietly for 3 minutes, participants began cycling while wearing their masks to introduce the Cardiopulmonary Exercise Test (CPET). After warming up, participants completed a continuous ramped cycling protocol with stages lasting 1 min. CPET familiarization was terminated at 75% of the estimated heart rate reserve (HRR).

#### 2.2.2 Visit 2: Cardiopulmonary Exercise Testing and Body Composition

Body composition was assessed using dual-energy X-ray absorptiometry (DXA: Discovery W, Hologic, Inc., Bedford, MA). The machine was calibrated prior to each use, and all scans were analyzed by the same technician before and after the exercise intervention. Participants then completed the CPET. The CPET consisted of a ramp cycling protocol of a 5-minute warm-up with no resistance, followed by 3 minutes at 20 watts (W) before 15 W increments applied each minute until volitional exhaustion. Breath by breath metabolic analysis was assessed using a Parvo Medics TrueMax 2400 Metabolic System (Parvo Medics, Salt Lake City, UT).

#### 2.2.3 Visit 3: Acute Exercise Testing

At least 24 hours after the CPET, participants returned to the laboratory having avoided caffeine, alcohol, and exercise in the previous 12-24 hours. After 10 minutes of supine rest, an indwelling venous catheter was inserted into the antecubital vein of the forearm, and a resting blood sample was obtained (Pre). The catheter was periodically flushed with 0.9% sodium chloride saline. The acute exercise trial was 60% of the peak power calculated from the CPET (27). Following a 1-minute warm-up at 0% and 1-minute at 30% of peak power, 10 x 3-minute intervals at 60% peak power interspersed with 1.5 minutes of active recovery were completed. HR and RPE were determined in the last 30 seconds of each stage. Additional blood samples were obtained immediately following exercise (Post) and after 1 hour of seated recovery with ad libitum access to water (1Hr Post).

### 2.3 Exercise Training Intervention

Exercise training consisted of 3 x 1-hour semi-supervised sessions/week for 16 weeks at the “Get REAL and HEEL Breast Cancer Rehabilitation Facility” (25). **Table 1** shows that of the prescribed 48 sessions, participants attended 35.6 ± 10.6 days (74.2 ± 22.0%). Participants completed 24.3 ± 11.2 days (50.7 ± 23.4%) at the prescribed aerobic intensity and duration. Participants completed 12.9 ± 5.5 days (26.8 ± 11.5%) at the prescribed resistance training intensity at the workload.

### 2.4 Hematology Analyses

#### 2.4.1 Blood Counts

Immediately following each blood collection, complete blood counts were performed in duplicate and averaged with a maximal white blood cell difference of 0.1 cells/µL as previously described (28) (Sysmex XP-300, Lincolnshire, IL, USA).

### 2.5 Neutrophil Bactericidal Functions

Phagocytosis of opsonized FITC-labelled *E.coli* (ThermoFisher Scientific, USA) was assessed in heparin-treated fresh whole blood as previously described (16). Briefly, 100 µL of blood was incubated for exactly 10 minutes at 4°C (control) or 37°C (test) with FITC-labelled *E.coli*. Phagocytosis was halted by the addition of cold phosphate-buffered saline (PBS), whilst cell surface-bound FITC was quenched by the addition of 1% trypan blue solution. Unbound-free bacteria were removed by washing in 4°C PBS, and erythrocytes lysed, and leukocytes fixed using 1% fix/lyse solution (ThermoFisher Scientific). Cell DNA was counterstained by addition the of propidium iodide (PI) before flow cytometry analysis was performed.

Reactive oxygen species (ROS) oxidative burst was assessed in heparin-treated whole blood that was incubated for exactly 10 minutes at 37°C with opsonized unlabeled *E.Coli* (test) or PBS (control). Following incubation, 2.5 µg/mL dihyrdorhodamine-123 (DHR-123), which is converted to fluorescent rhodamine-123 (R-123) in the presence of reactive oxidants, was added for exactly 10 minutes at 37°C. Oxidative burst was halted by the addition of erythrocyte lysis/leukocyte fixation buffer before washing and leukocyte DNA stained prior to flow cytometry analysis.

### 2.6 Cell Surface Receptor Expression

Neutrophil surface receptor expression was assessed in heparin-treated fresh whole blood similar to previously described (18). Briefly, 100µL of heparin-treated blood was dispensed into 5mL tubes at 4°C in the dark. Cells were stained with anti-CXCR2-PE (ThermoFisher, clone 5E8-C7-F10), anti-CD16-FITC (BD Bioscience, clone 3G8), anti-TLR2-Alexa-647 (BD Bioscience, clone 11G7), and anti-TLR4-APC (ThermoFisher, clone HTA-125) or their relevant concentration-matched isotype control for 30 minutes at 4°C in the dark. Following incubation, cells were washed twice in at 4°C PBS and erythrocytes lysed, and leukocytes fixed using 1% fix/lyse solution (ThermoFisher Scientific). Following fixation, cells were washed twice and resuspended in 300µL PBS/1%BSA for analysis by flow cytometry.

### 2.7 Flow Cytometry Assessment

All flow cytometry analyses were conducted on a BD FACSCanto II (BD Bioscience, USA) flow cytometer equipped with 3-lasers (Red [633 nm HeNe], Blue [488 nm solid state], and Violet [405 nm solid state]. This flow cytometer was part of the Duke Cancer Institutes flow cytometry core facility and was maintained with daily QC bead assessment and regular laser alignment to ensure reliability of sample assessment across time. The gating strategy (**Supplementary Figure 1**) involved 1) exclusion of doublets by FSC A v FSC H; 2) This gate was then used for identification of cell subtypes by FSC H v SSC H. In whole blood samples this allows for clear discrimination between lymphocytes, monocytes and neutrophils. A gate was placed around the neutrophil population; 3) following compensation, single histograms of surface markers were gated from neutrophils. 100% of neutrophils expressed each of the surface markers. Compensation of samples (e.g. FITC v PI/PE) were standardized across all samples. Phagocytic and oxidative burst were determined by the relative increase in median fluorescence intensity (MFI) compared to negative controls. Similarly, surface receptor expression were determined by the relative increase in MFI compared to negative controls. Data were analyzed using FCS Express 6 (FCS Express, USA).

### 2.8 Plasma Analyses

EDTA treated blood samples were placed on ice immediately after venipuncture. Samples were centrifuged at 3000 x *rpm* and samples stored at -80°C until analyzed. Plasma samples were analyzed only for IL-8 and IL-6 concentrations. Concentrations were determined in duplicate using a human pro-inflammatory sandwich immunoassay according to the manufacturer’s instructions (Meso Scale Discovery, Rockville, MD). Within plate standards R^2^=.999 and between plate standard R^2^=.989. The lower limits of detection (LLOD) were IL-6 (0.11 pg/mL) and IL-8 (0.08 pg/mL). All samples had concentrations greater than the LLOD.

### 2.9 Statistical Analyses

We completed the statistical analyses similar to previously described using IBM SPSS version 26.0 (Armonk, NY, USA), with all data presented as mean (standard deviation) unless otherwise stated (18, 26). The primary outcome of this sub-study was the exercise training mediated change in resting neutrophil phagocytic capacity. We powered the study to detect a 15% increase from Base to Final in phagocytic capacity based on our previous study, which suggested a minimum of 13 participants (17). We used descriptive statistics to summarize participant characteristics at Base with differences between BCS and Controls assessed by independent T-tests. At rest, we completed pairwise comparisons of BCS variables using paired T-tests, and we used independent T-tests to compare Controls to the BCS group (18). Control variables’ mean values were considered 100% to reflect the appropriate age and fitness response, while the BCS mean values are presented as the percentage of Controls. We examined acute immune analyses using a linear mixed model, with group and time as fixed factors and participants as a random effect. Our analyses consisted of Base BCS v Controls, Base BCS v Final BCS, and Final BCS v Controls to provide our readers with the most informative data. Results are presented with 95% confidence intervals (CI) and effect sizes calculated as Hedges G (g), such that the values of 0.2, 0.5, and 0.8 represent small, medium, and large differences. Relationships between adherence or compliance to the exercise program and physiological and neutrophil changes were assessed with Spearman’s correlations. We report unadjusted T-test p-values, and significance accepted as p ≤ 0.05.

## 3 Results

### 3.1 Demographics and Clinical Characteristics

Demographics and clinical characteristics are presented in **Table 2** for all the women who completed the study. BCS were similar to Control women for all demographics, except for height (p=0.005). BCS were 0.4 – 7.8 months from final treatment before beginning the study. Most women were stage 2 (50%), grade 3 (50%), estrogen receptor (ER)+ (82%), progesterone receptor (PR)+ (69%), and human epidermal growth factor receptor 2 (HER2)^neg^ (75%). Treatments included lumpectomy (75%), radiation (89%), chemotherapy (75%), taxane (63%), and anthracycline (19%).

**Table 2:** Participant demographics, cancer characteristics and physical fitness at baseline.

	Controls (n=11)	BCS (n=16)	P value	95% C.I.	*g*
**Demographics**					
Age (yrs.)	53 (10)	56 (11)	0.402	(-4.9, 11.9)	.287
Caucasian [N (%)]	9 (82)	13 (82)			
Height (cm)	157.8 (3.8)	165.5 (7.7)	**0.005**	(2.5, 12.9)	1.197
Weight (kg)	69.7 (12.7)	74.5 (12.6)	0.338	(-5.4, 15.0)	.380
BMI (kg/m^2^)	27.4 (3.9)	27.4 (5.6)	0.978	(-4.0, 4.1)	.000
Blood Pressure (mmHg)					
Systolic	134 (16.5)	127 (13.3)	0.253	(-18.6, 5.1)	.478
Diastolic	80 (7.8)	79 (7.4)	0.682	(-7.3, 4.9)	.132
MAP	97 (10.4)	94 (9.1)	0.376	(-11.2, 4.4)	.311
Postmenopausal [N (%)]	7 (64)	10 (63)			
**Cancer Characteristics**					
EOT to Enrollment in Months		2.8 (2.3)			
Stage [N (%)]					
1		4 (25)			
2		8 (50)			
3		4 (25)			
Grade [N (%)]					
1		3 (19)			
2		5 (31)			
3		8 (50)			
ER+ [N (%)]		13 (82)			
PR+ [N (%)]		11 (69)			
HER2^neg^ [N (%)]		12 (75)			
Surgery (lump/mast)		12/4			
Radiation [N (%)]		14 (88)			
Anti-HER2 [N (%)]		5 (31)			
Chemotherapy [N (%)]		12 (75)			
Taxane [N (%)]		10 (63)			
Anthracycline [N (%)]		3 (19)			

BCS, Breast Cancer Survivors; BMI, Body Mass Index; MAP, Mean Arterial Pressure; EOT, End of Treatment; ER, Estrogen Receptor; PR, Progesterone Receptor; HER2, Human Epidermal Growth Factor Receptor-2; 6MWT, 6-Minute Walk Test; TUG, Timed-Up-and-Go. Data are mean (SD) unless otherwise indicated. Hedges G (g) represents the effect size, such that the values of 0.2, 0.5, and 0.8 represent small, medium, and large differences.

Bold numbers represent signifcant differences.

### 3.2 Physiological Fitness and Function

**Figure 1:**
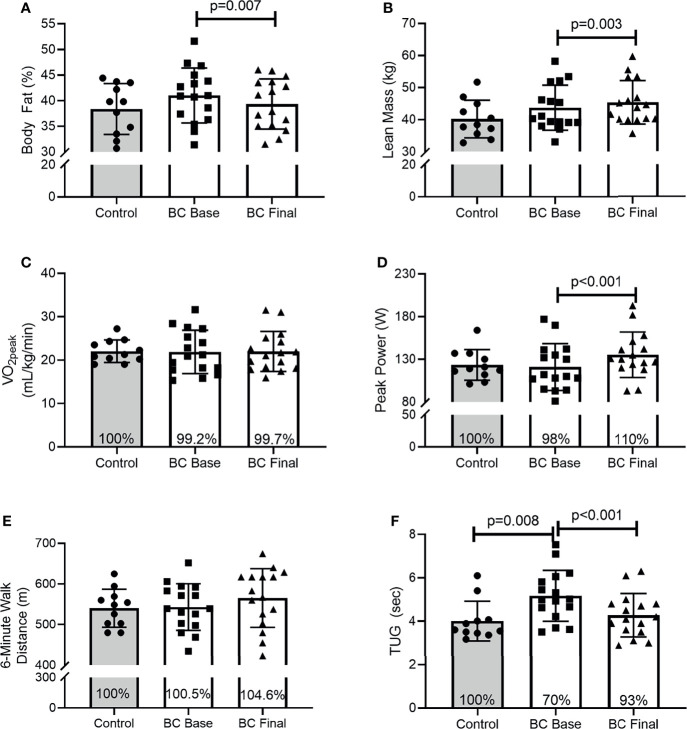
Body composition, physical and functional fitness between Controls and BCS measured once in Controls, and before and after 16 weeks of exercise training in BCS. **(A)** Body fat percentage, **(B)** Lean mass, **(C)** relative cardiorespiratory fitness (VO2peak), **(D)** peak power during CPET, **(E)** 6-minute walk distance, and **(F)** Timed-up-and-go test. Where shown, the Controls mean values have been assigned 100% to represent the optimal response, and BCS the percentage of Control means. Data are mean (SD).

Results are presented as [95% CI (x,y), p-value]. At Base, BCS and Control women were similar for body fat percentage [**Figure 1A**: (-1.564, 6.825), p=0.208] or lean mass [**Figure 1B**: (-3.971, 4.323), p=0.931], aerobic capacity [**Figure 1C**: (-3.565, 3.217), p=0.917], and peak power output [**Figure 1D**: (-21.874, 17.022), p=0.799], and 6-minute walk distance [**Figure 1E**: (-40.309, 45.865), p=0.895]. In contrast, Control women completed the timed-up-and go faster than BCS [**Figure 1F**: (.291, 2.030), p=0.008]. Following the intervention, BCS reduced their body fat percentage [**Figure 1A**: (.537, 2.825), p=0.007] and increased their lean mass [**Figure 1B**: (-2.825, -.538), p=0.003]. As such, BCS lean mass post-intervention was marginally higher than Control women’s were (p=0.058). Following the intervention, BCS aerobic capacity was unchanged [**Figure 1C**: (-1.675, 1.446), p=0.878], but peak power increased [**Figure 1D**: (-20.010, -8.739), p<0.001]. Although the 6-minute walk distance did not improve [**Figure 1E**: (-49.843, 5.265), p=0.105], TUG time decreased [**Figure 1F**: (.232, 1.547), p=0.011] and was no longer different from Control women (**Figure 1E**: p=0.482).

### 3.3 Neutrophils Functions

**Figure 2:**
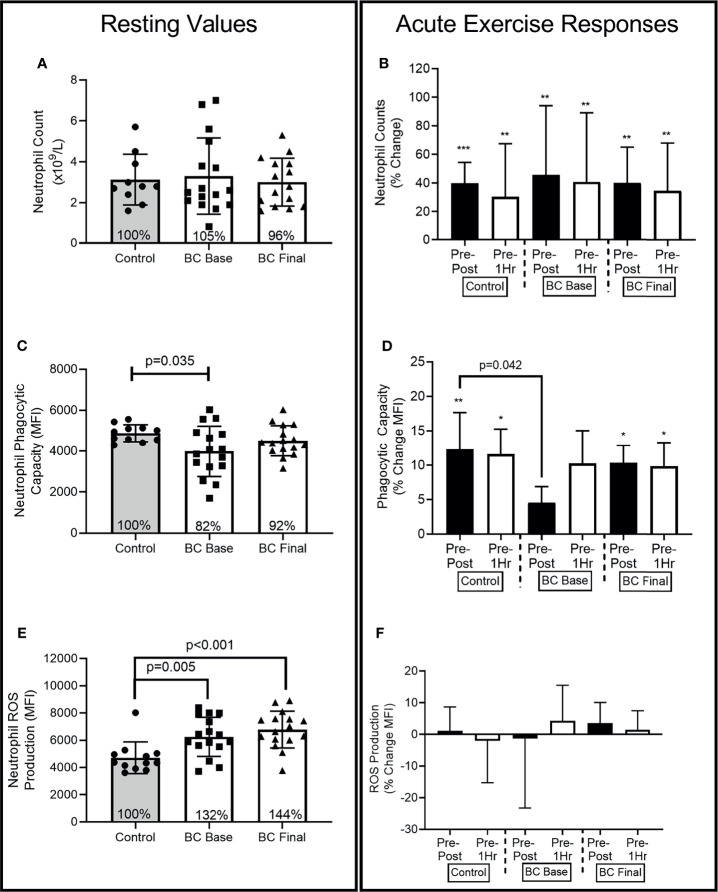
Neutrophil differences between Controls and BCS measured once in Controls, and before and after 16 weeks of exercise in BCS. Panels show, resting values (Left Side) and acute exercise responses (Right Side) for percentage changes pre exercise to immediately post exercise (Pre-Post), and Pre exercise to 1 hour after exercise completion (Pre-1Hr). **(A)** Neutrophil absolute counts measured once in Controls, and before and after 16 weeks of exercise in BCS. **(B)** Neutrophil percentage changes in response to acute exercise in Controls, and before and after 16 weeks of exercise in BCS. **(C)** Neutrophil phagocytic capacity for E.coli **(D)** Neutrophil phagocytic capacity percentage changes in response to acute exercise. **(E)** Neutrophil ROS production for E.coli **(F)** Neutrophil ROS production percentage changes in response to acute exercise. *p < 0.05, **p < 0.01 and ***p < 0.001 significant change for groups acute response. Where shown, the Controls mean values have been assigned 100% to represent the optimal response, and BCS the percentage of Control means. Data are mean (SD).

**Figure 3:**
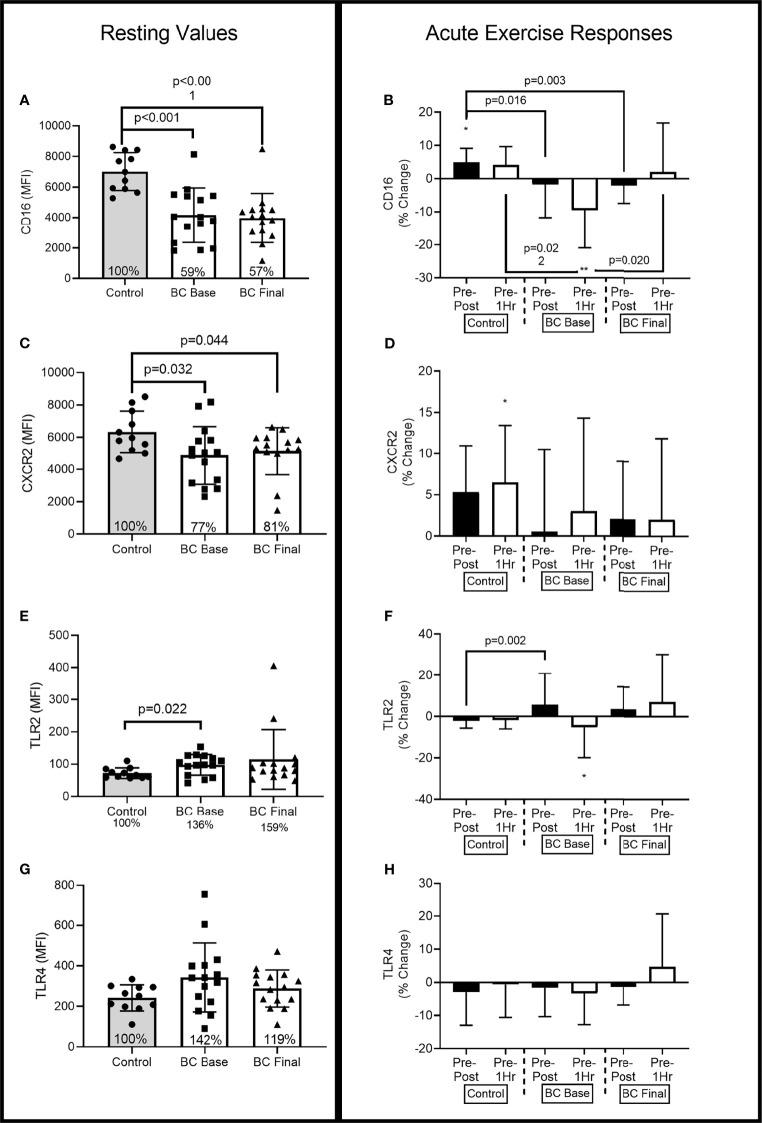
Neutrophil surface receptor expression (MFI) differences between Controls and BCS measured once in Controls, and before and after 16 weeks of exercise in BCS. Panels show, resting values (Top Panels) and acute exercise responses (Bottom Panels) for percentage changes pre exercise to immediately post exercise (Pre-Post), and Pre exercise to 1 hour after exercise completion (Pre-1Hr). Panels show CD16 expression at rest **(A)** and during acute exercise **(B)**, CXCR2 expression at rest **(C)** and during acute exercise **(D)**, TLR2 expression at rest **(E)** and during acute exercise **(F)**, and TLR4 expression at rest **(G)** and during acute exercise **(H)**. *p < 0.05, and **p < 0.01 significant change for groups acute response. Where shown, the Controls mean values have been assigned 100% to represent the optimal response, and BCS the percentage of Control means. Data are mean (SD).

**Figure 4:**
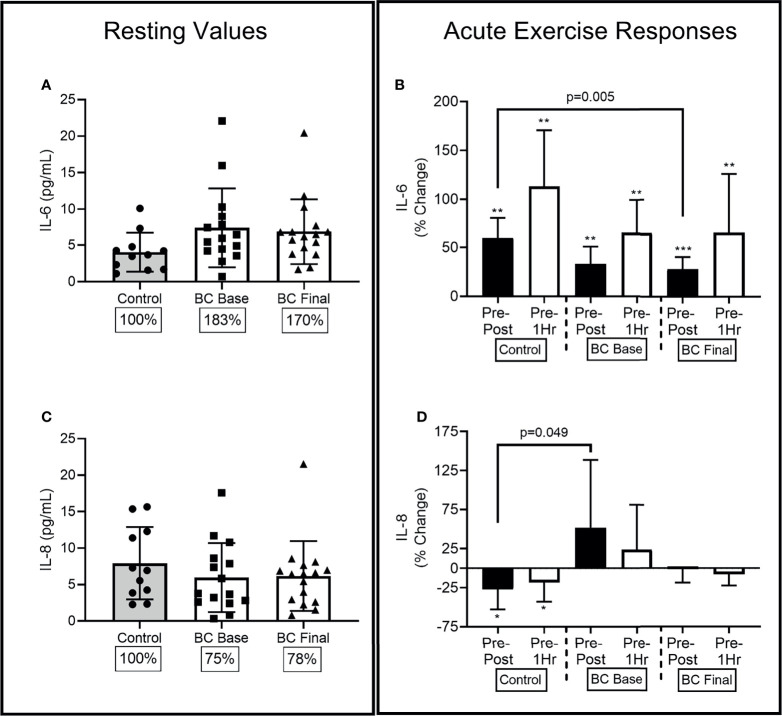
Circulating serological IL-6 and IL-8 differences between Controls and BCS measured once in Controls, and before and after 16 weeks of exercise in BCS. Panels show, resting values (Left Panels), and acute exercise responses (Right Panels) for percentage changes pre exercise to immediately post exercise (Pre-Post), and Pre exercise to 1 hour after exercise completion (Pre-1Hr). Panels show IL-6 concentrations at rest **(A)** and during acute exercise **(B)**, and IL-8 concentrations at rest **(C)** and during acute exercise **(D)**, *p < 0.05, **p < 0.01 and ***p < 0.001 significant change for groups acute response. Where shown, the Controls mean values have been assigned 100% to represent the optimal response, and BCS the percentage of Control means. Data are mean (SD).

**Figures 2**–**4** show the differences for Base and Final BCS values and Base Control women (Left Panel). The acute exercise responses at each time point (Right Panel) are representative of the percentage changes from pre-exercise to immediately post-exercise (Pre-Post) and from pre-exercise to 1 hour after exercise completion (Pre-1Hr Post).

#### 3.3.1 Resting Values for Neutrophil Counts

At Base, BCS and Controls had similar absolute neutrophil counts (**Figure 2A**) [ANC: (-1.134, 1.774), p=0.801], while exercise training did not change BCS resting ANC.

#### 3.3.2 Acute Exercise Responses

At Base (**Figure 2B**) (F=23.63, p<0.001, η^2^=.518) and Final (F=30.57, p<0.001, η^2^=.617), there were main effects for acute exercise time. Both Controls and BCS increased ANC Pre-Post, with ANC remaining elevated 1 hour after exercise completion.

#### 3.3.3 Resting Values for Phagocytosis

At Base (**Figure 2C**), BCS phagocytic capacity was approximately 18% lower than Control women’s were [(-1733.579, -66.673), p=0.035], while exercise training marginally increased BCS resting phagocytic capacity so that it was no longer lower than Controls [(-876.711, 203.360), p=0.117].

#### 3.3.4 Acute Exercise Responses

At Base (**Figure 2D**), there was a main effect for acute exercise time (F=7.55, p=0.02, η^2^=.256) with only Controls increasing by 12.3% (SD 17.7%) from Pre-Post [(-957.342, 193.567), p=0.005] and Pre-1Hr [(-922.804, -193.567), p=0.005]. BCS Pre-Post response was 12% (26%) lower than Controls at Base [(-23.249, -.491), p=0.042] but no longer different than Controls at Final [(-16.914, 7.266), p=0.418].

#### 3.3.5 Resting Values for ROS Production

BCS ROS production was approximately 32% and 44% higher than Controls at Base [(241.325, 2676.447), p=0.005] and Final [(826.117, 3188.369), p<0.001], while exercise training did not change BCS resting ROS production (p=0.974) (**Figure 2E**).

#### 3.3.6 Acute Exercise Responses

There were no effects for acute exercise time at Base (F=0.00, p=0.999, η^2^=.000) or Final (F=1.80, p=0.177, η^2^=.076) (**Figure 2F**). There were also no group x time interactions.

### 3.4 Neutrophils Receptor Expression

#### 3.4.1 CD16 and CXCR2

##### 3.4.1.1 Resting Values for CD16 Expression

At Base, BCS neutrophil CD16 expression was approximately 41% lower than Controls [**Figure 3A**: (-3754.187, -1252.219), p<0.001] and remained lower following 16 weeks of exercise training [**Figure 3A**: (-4095.618, -1594.788), p<0.001].

##### 3.4.1.2 Acute Exercise Responses

At Base, there was a group x time interaction (F=9.90, p<0.001, η^2^=.310), that was less evident following 16 weeks of training (F=2.73, p=0.076, η^2^=.110) (**Figure 3B**). At Base BCS Pre-Post responses were 12% (21%) lower than Controls [(-20.089, -2.341), p=0.016] while Pre-1Hr responses were 13% (25%) lower than Controls [(-23.453, -2.074), p=0.022]. Following training, BCS Pre-Post responses were 9% (15%) lower than Controls [(-15.263, -3.440), p=0.003], while Pre-1Hr responses were no longer different than Controls [(-20.990, 6.679), p=0.294]. As compared to Final responses, BCS Base CD16 Pre-1Hr was 12% (16%) lower [(-21.060, -2.138), p=0.020].

##### 3.4.1.3 Resting Values for CXCR2 Expression

At Base, BCS expression of CXCR2 was approximately 23% lower than Controls (**Figure 3C**) [(-2702.014, -80.993), p=0.032] and remained lower following 16 weeks of exercise training [(-2391.912, -147.451), p=0.044].

##### 3.4.1.4 Acute Exercise Responses

At Base, there was a main effect for acute exercise time (F=3.32, p=0.045, η^2^=.131) with Control women increasing by 6% (5%) from Pre-Post [(-651.878, 34.059), p=0.075] and by 7% (7%) from Pre-1Hr [(-688.566, -39.615), p=0.030]. BCS CXCR2 expression was not altered by acute exercise at Base or following 16 weeks of exercise but was also not different from Controls.

#### 3.4.2 TLR2 and TLR4 Expression

##### 3.4.2.1 Resting Values for TLR2 Expression

At Base, BCS expression of TLR2 was approximately 36% higher than Controls [(5.004, 43.466), p=0.022], while 16 weeks of exercise training did not change BCS TLR2 expression (**Figure 3E**).

##### 3.4.2.2 Acute Exercise Responses

At Base, there was a main effect for acute exercise time (F=3.84, p=0.029, η^2^=.155) and a group x time interaction (F=3.93, p=0.027, η^2^=.158) (**Figure 3F**). At Base, BCS TLR2 expression reduced by 5% (15%) from Pre-1Hr [(1.662, 12.005), p=0.012], while the Pre-Post responses were 15% (4%) higher for BCS compared to Controls [(5.831, 23.585), p=0.002].

##### 3.4.2.3 Resting Values for TLR4 Expression

At Base, BCS expression of TLR4 was 42% higher, albeit not significant, than Controls [(-30.403, 239.91), p=0.074], while 16 weeks of exercise training did not change BCS TLR4 expression (**Figure 3G**).

##### 3.4.2.4 Acute Exercise Responses

Acute exercise did not change TLR4 expression in either BCS or Controls, and 16 weeks of exercise training did not alter this response (**Figure 3H**).

### 3.5 Cytokine Levels

#### 3.5.1 Resting Values for IL-6 Levels

At Base, BCS IL-6 levels were marginally higher than Controls [(-.707, 7.352), p=0.079], while 16 weeks of exercise training did not change BCS resting IL-6 levels (**Figure 4A**).

#### 3.5.2 Acute Exercise Responses

At Base, there was a main effect for acute exercise time (F=20.04, p<0.001, η^2^=.477), with responses similar between the two groups. Base increases were observed Pre-Post for Controls (58% (32%), [-3.154,.878], p=0.004) and BCS [34% (34%), (-2.834, -.911), p=0.002], and remained elevated Pre-1Hr for Controls [107% (75%), (-5.175, -1.243), p=0.008] and BCS [70% (74%), (-4.799, -1.158), p=0.002] (**Figure 4B**). Following the training intervention, BCS still had increases Pre-Post [(-2.936, -1.158), p<0.001] and Pre-1Hr [(-5.467, -1.776), p=0.002], but the Pre-Post was 35% (87%) higher for Controls compared to BCS [(11.447, 57.652), p=0.005].

#### 3.5.3 Resting Values for IL-8 Levels

At Base, BCS and Controls had similar IL-8 levels [(-5.804, 2.721), p=0.461], while 16 weeks of exercise training did not change BCS resting IL-8 levels (**Figure 4C**).

#### 3.5.4 Acute Exercise Responses

At Base, there was a main effect for acute exercise time (F=3.82, p=0.029, η^2^=.461) and a group x time interaction (F=3.52, p=0.038, η^2^=.133) (**Figure 4D**). Control women’s IL-8 levels reduced by 27% (38%) from Pre-Post [(.515, 4.055), p=0.014] and by 18% (37%) from Pre-1Hr [(.391, 3.695), p=0.018] resulting in the Pre-Post difference between BCS and Controls at Base [(.571, 198.30), p=0.049]. Following 16 weeks of training the Pre-Post difference between BCS and Controls were no longer different [(-4.010, 54.889), p=0.087].

### 3.6 Correlations

Higher adherence to the program was associated with larger changes in peak power during the CPET (r^2^=.534, p=0.033), but no other physiological outcomes. Higher adherence to the program was associated with larger positive changes in resting CD16 expression (r^2^=.552, p=0.033), negative changes in TLR4 expression (r^2^=-.532, p=0.041) and marginally larger positive changes in resting phagocytic capacity (r^2^=.493, p=0.053). Higher adherence was also associated with larger positive changes in acute exercise Pre-Post responses for CD16 expression (r^2^=.517, p=0.049) and TLR2 expression (r^2^=.576, p=0.025).

## 4 Discussion

Neutrophils are the first immune cell to respond against infectious organisms, and breast cancer survivors are at an increased risk of infections due to treatment-related immune suppression (3, 29). Although exercise training reduces many treatment-related side effects (e.g., fatigue) (30), less is known for neutrophil functional responses. We observed BCS likely have dysregulated neutrophil bactericidal responses indicative of breast cancer-specific intrinsic neutrophil defects. Further, dysregulated responses were evident during an acute bout of exercise, while 16 weeks of exercise training partially restored these responses, reflective of levels observed in healthy women. We characterized the dysregulated functional responses with lower cell surface expression of CD16, CXCR2, and higher TLR2 expression. Changes in IL-8 levels and expression of CD16 and TLR2 had differential responses to acute exercise at Base that were partially restored following exercise training.

### 4.1 Resting Neutrophil Functional Differences Between BCS and Healthy Women

Neutrophils play a complex role in breast cancer, including antitumor and tumor promotion, and are predictive of prognosis (31, 32). Neutrophil functions are diverse and include reduced chemotaxis, phagocytosis, and ROS-mediated oxidative microbial killing (33). Impaired functions may result from treatments, including anthracyclines, alkylating agents, or taxanes, disrupting cell cytoskeletal formation and membrane compliance (4, 5, 34). Although it is unclear how long this lasts, other innate and adaptive immune cells have impaired functions for approximately 12 months following therapeutic completion (2, 3). Coupled with an increased risk of infections, BCS neutrophil dysfunctions may also last for extended periods (2–4). Here, we show that BCS and healthy women have similar resting absolute neutrophil counts (ANC). However, BCS neutrophils are phagocytosing fewer bacteria while producing higher amounts of intracellular ROS to kill fewer bacteria. Because ROS production typically increases linearly with more ingested bacteria (35), it is unclear what this observation means. In the tumor microenvironment, neutrophils release more extracellular ROS resulting in a pro-tumorigenic response and immune suppression (36), but ROS release can also kill tumor cells (32). ROS are also involved in other cellular processes, including cell signaling and neutrophil apoptosis (37). Increased neutrophil apoptosis is associated with reduced expression of CD16 (38), while low CXCR2 expression is associated with increased neutrophil homeostatic clearance (39–41). As we observed lower expression of CD16 and CXCR2 on BCS neutrophils, it is plausible that more BCS neutrophils are prone to apoptosis and homeostatic clearance. In effect, the ratio between newly released neutrophils from the bone marrow and old circulating neutrophils may be different in BCS. These findings agree with others that have observed poor neutrophil characteristics during and following breast cancer therapy (4, 5). Further work is required to determine the extent of neutrophil dysfunction in BCS, including a more comprehensive phenotype and functional characterization.

### 4.2 Exercise Training Induced Neutrophil Functional Responses

Few studies that we are aware of have examined the role of exercise training on specific neutrophil functions in breast cancer patients (13, 42–45) – all but one assessed ANC changes. However, counts do not accurately reflect cellular processes that protect against infection (46). Our results are similar to Fairey and colleagues who assessed mitogen-stimulated ROS production before and after 15 weeks of exercise training (13). Here, we used a bacterial challenge requiring all bactericidal processes to occur in sequence and found no effect for exercise training on ROS production. We are unaware of any studies using exercise training to modify neutrophil bacterial phagocytosis or surface receptor expression in BCS. We observed that 16 weeks of exercise training resulted in no significant changes for resting phagocytosis of bacteria or expression levels of CD16, CXCR2, TLR2, or TLR4. In adults with autoimmune and metabolic diseases, neutrophil functions can be increased following 10 weeks of high-intensity interval training that promotes increased cardiorespiratory fitness (18, 19). As cardiorespiratory fitness did not increase in our study, it is plausible that neutrophil functional changes require higher intensities of exercise coupled with broader physiological responses. Alternatively, exercise training may not be capable of improving resting neutrophil functions in BCS. Future studies should aim to determine the dose and intensity of exercise required to improve pertinent neutrophil functions with a goal to reduce the risk of infections.

### 4.3 Neutrophils and Acute Exercise

In healthy adults, the immunological, inflammatory, and endocrine responses to single bouts of acute exercise have been relatively well-characterized (47). During exercise, the mobilization of effector immune cells (i.e., NK-cells, CD8+ T-cells, and neutrophils), coupled with changes in inflammatory cytokine levels and hormones, is suggested to be an appropriate stress response (8). Here, we show the ANC responses to acute exercise are similar between BCS and healthy women, and 16 weeks of exercise training does not change the response. These findings are comparable to other studies that observed no differences for NK-cell responses to acute exercise in BCS (24) or prostate cancer survivors (27) compared to healthy controls. However, we now show for the first time that neutrophil phagocytic and ROS responses to acute exercise may be dysregulated in BCS, and 16 weeks of exercise training partially restores some of these functions. Specifically, during and after acute exercise at Base, BCS phagocytosis is lower, while ROS production contrasts that of healthy women’s. Following 16 weeks of exercise training, BCS phagocytosis is partially restored to similar levels observed in healthy women. These effects at Base coincide with differential BCS neutrophil CD16 expression responses, responses that are partially restored following 16 weeks of exercise training. Our observation that the Pre-1Hr CD16 response was the most influenced by exercise training, and not the Pre-Post response, suggests the CD16 acute response is delayed rather than dysfunctional. It is unclear why this would be, our correlation data suggests that better adherence to exercise is associated with higher resting CD16 expression and a larger Pre-Post response, indicating that more exercise is needed to elicit CD16 changes. Both exercise training and acute exercise had little effect on TLR2 and TLR4 expression. Both TLRs are expressed on all neutrophils, with TLR4 typically expressed less than TLR2. Increased expression of both TLRs occur upon stimulation and activation of neutrophils with bacterial peptides and GM-CSF (48). Unlike monocytes, neutrophil functions are heavily dependent on TLR2 mediated pathways, including for typical TLR4 ligands such as LPS based lipopeptides (48). We do not know if exercise influenced GM-CSF levels, and we are unable to measure TLRs during an *E.coli* challenge. Others have shown that GM-CSF is unchanged during acute exercise (49), and may be why so little effects were observed for TLR2 and TLR4. Although neutrophil TLR4 and CD14 identify *E.coli*, the process of whole blood phagocytosis is equally dependent on FCγ receptors, including CD16a (FCγRIIIa) (50–52). As such, it appears that BCS neutrophils may be similarly capable of TLR mediated recognition of bacteria as healthy women are. Still, because of the dysregulated CD16 responses, they are likely to phagocytose bacteria less well. Given neutrophil functions are impaired in BCS (4, 5, 34), it remains unknown whether our observed exercise-training acute effects translate to a lower risk of infections.

### 4.4 Inflammatory Cytokine Responses

Neutrophils are pro-inflammatory immune cells, and functional responses stimulated by cytokines, including IL-6 and IL-8 (6). Further, these cytokines tend to be higher in breast cancer patients, and lower levels during chemotherapy are associated with better outcomes (53–55). However, there is conflicting data regarding IL-6 and IL-8 responses to exercise training in cancer survivors, including BCS (56, 57). Specifically, in two meta-analyses, Meneses-Echávez and colleagues suggested that exercise training reduced IL-6 and IL-8 in BCS (56). In contrast, Khosravi and colleagues suggested there were no changes for IL-6 or IL-8 for mostly BCS (N=17), but also prostate (N=5), colorectal (N=1), lung (N=1), stomach (N=1), and germ cell (N=1) (57). The inclusion of cancers other than breast cancer may have influenced the findings for IL-6 and IL-8, but Khosravi did show that CRP and TNF were more sensitive to exercise changes. Here, we observed marginally higher resting IL-6 and similar resting IL-8 levels in BCS as compared to Control women, while exercise training did not change levels. Similar to exercise studies in the general population, the effects on individual cytokine levels are complex, with many factors including the physiological state of participants and the exercise intensity/mode/duration and adherence influencing results (58, 59).

In terms of acute exercise, the only study we are aware of in younger BCS provoked a 210% increase for IL-6 and a 20% increase for IL-8 levels immediately after 2-hours of extremely demanding exercise (60). Here, using a shorter and less challenging protocol, we show BCS had a significant 33% increase for IL-6 and no significant change for IL-8 following 45-minutes of acute exercise. However, BCS Base IL-8 responses contrasted healthy women and resulted in a group difference at Base, suggestive of a BCS dysregulated acute exercise response (59, 61). Upon completing our exercise-training program, the BCS acute exercise IL-8 response appeared more similar to healthy women, suggesting that exercise partially restored this response.

### 4.5 Limitations and Future Directions

A significant strength of our study is that we assessed neutrophil responses to acute exercise before and again after the 16-week training intervention. These factors are typically independently evaluated and do not reflect the true extent of exercise training-induced changes. We also report neutrophil counts, phenotypes, and functions that can be used for comparison with other studies. The inclusion of age- and physical activity-matched healthy control group provides insight into the normal immune response and whether exercise training moves responses in the correct direction. Our study is also not without limitations. We were unable to characterize comprehensive neutrophil subsets, including low- (immature) and high- (mature) density neutrophils or the presence of neutrophil-myeloid-derived suppressor cells. Although the phenotype of these cells is complex, they have distinct functional differences. We did not have a BCS non-exercise group. This limits our ability to confirm the degree of the immune and inflammatory effects because of the time since completing treatment or the exercise intervention. Our correlative data indicates that exercise adherence is associated with better changes in CD16 expression and phagocytosis, suggesting that exercise rather than time since treatment is influencing neutrophil functions. As such, given the ethical dilemma of a BCS control group and how other innate immune cells take approximately 12 months to recover (2, 3), we are confident that four months of exercise training positively influences immune cell functional recovery. Finally, this study is an exploratory assessment of neutrophil functions in BCS and until a larger randomized control trial is completed, the interpretation of results remain exploratory.

## 5 Conclusion

In conclusion, BCS demonstrated a dysregulated neutrophil bactericidal response at both rest and during acute exercise. Dysregulated functional responses were associated with differential resting and acute exercise responses for neutrophil expression of CD16, CXCR2, and TLR2 and serological levels of IL-8. Despite a lack of increase in cardiorespiratory fitness, 16 weeks of exercise training partially enhanced BCS neutrophil and IL-8 responses to similar levels observed in healthy women. With BCS at an increased risk of infections, neutrophils are responsive to exercise training. Whether this translates to a reduced risk of infections still needs to be established. Future studies should consider comparing healthy matched women and BCS neutrophil and immune responses to acute and chronic exercise in combination. This will help to tease out how exercise improves immunity in the breast cancer continuum.

## Data Availability Statement

The datasets generated and/or analyzed during the present study are not publicly available, owing to the risk of disclosure or deduction of private individual information, but they are available from the corresponding author on reasonable request.

## Ethics Statement

The studies involving human participants were reviewed and approved by Protocol Review Committee and the Institutional Review Board at the University of North Carolina at Chapel Hill. The patients/participants provided their written informed consent to participate in this study.

## Author Contributions

DB and EDH: conceptualization, methodology, investigation, formal analysis, writing-original draft, supervision. JL: methodology, investigation, writing-review and editing. CW: methodology, investigation, writing-review and editing. SS: investigation, writing-review and editing. EPH: investigation, writing-review and editing. LB: investigation, writing-review and editing. MA: investigation, writing-review and editing. DA: investigation, writing-review and editing. AD: formal analysis, writing-review and editing. BJ: investigation, writing-review and editing. GM: investigation, writing-review and editing. MD: investigation, writing-review and editing. HM: conceptualization, writing-review and editing, funding acquisition. KN: conceptualization, writing-review and editing. CB: conceptualization, methodology, investigation, writing-review and editing, supervision. All authors contributed to the article and approved the submitted version.

## Funding

This work was funded by the Breast Cancer Research Foundation (New York, NY) and other internal research funds.

## Conflict of Interest

The authors declare that the research was conducted in the absence of any commercial or financial relationships that could be construed as a potential conflict of interest.

## Publisher’s Note

All claims expressed in this article are solely those of the authors and do not necessarily represent those of their affiliated organizations, or those of the publisher, the editors and the reviewers. Any product that may be evaluated in this article, or claim that may be made by its manufacturer, is not guaranteed or endorsed by the publisher.
