# Supplementary material for: The Effects of 16 Weeks of Exercise Training on Neutrophil Functions in Breast Cancer Survivors
Source: Front Immunol. 2021 Oct 27;12:733101. doi: 10.3389/fimmu.2021.733101 (PMC8578958; doi:10.3389/fimmu.2021.733101)
Supplement: Supplementary file 1 [file DataSheet_1.pdf]

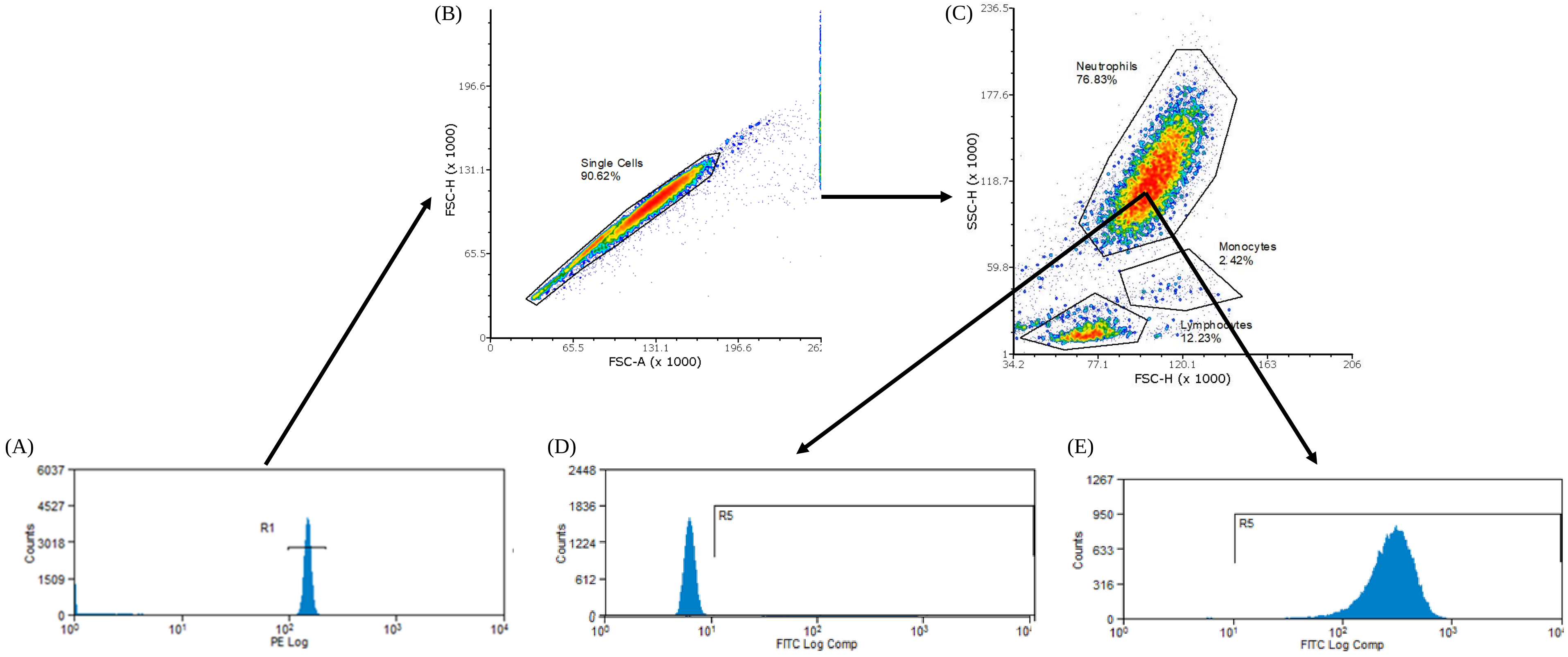

Supplementary Figure 1. Gating strategy for neutrophil analyses. Propidium iodide (PI) staining of all dead cells identifys the leukocytes required to assess phagocytosis and ROS (A). From R1, and for cell surface phenotyping, FSC-A v FSC-H discrimination of single and doublet cells is conducted (B). Single cells are gated, and this gate is applied to a FSC-H v SSC-H plot to identify specific populations of leukocytes (C). Cells of interest (i.e. neutrophils) are gated and following compensation of spectral overlap the negative/isotype control median fluorescence intensity (MFI) and the test/surface marker MFI are identified, with the test/phenotype MFI subtracted from the negative/isotype control.
